# Some General Influences of the War

**Published:** 1914-12-19

**Authors:** 


### SOME GENERAL INFLUENCES OF THE WAR.

The sufferings and miseries and horrors of war are more or less near to all of us. The public anxieties and private griefs which it inflicts can hardly be exaggerated. That such influences are in themselves hurtful no one can doubt, and least of all those whose ministry of public service is found in the practice of medicine. Strain, uncertainty, the loss of friends, doubts of the future—all these mean the play of agencies hurtful both to the individual and to the public health. Yet it is necessary to accept accomplished facts. However much we may deplore it, we are living under the actual stress of war. The evil consequences which this involves might well have stayed the hands of those who lit the flame, and their responsibility is not an enviable one. But the Rubicon has been crossed, and considerations which failed to arrest the decision have now to be accepted in the full range of their influence. Whatever our regret, the facts are now our masters. Starting from such a point we may perhaps ask whether in this disastrous evil there may not be found some elements of good. Not good which will compensate or atone for the evil, but which may yet be taken as at least possible in the circumstances. It were best that the whole chapter had never been written; but being written, is there anything which in some measure relieves its unhappiness? An attempt to find an affirmative answer is at least worthy of excuse.

The appeal of war, in history and in present fact, certainly addresses itself to qualities which have played no small part in the development and achievements of mankind. Courage, discipline, self-sacrifice for great aims, have been powerful forces in mental and moral development. Not only muscle but mind has felt the stimulating influence of armed struggle. In this way war has done something to promote the old ideal—*mens sana in corpore sano.* It is only necessary to look around to see that such an influence is still continued. The youth of the nation in large numbers is, under the stimulus of the existing crisis, cultivating bodily fitness and efficiency in a manner and to a degree unknown for several generations. The effect of such discipline on the individual can be seen in all our military camps and drill-grounds. The message is carried thence to countless homes by youthful soldiers who return for a few hours to the welcome of proud though anxious relatives. It is impossible to doubt that these multiplied experiences can fail to exercise a considerable influence on public opinion in relation to the value of personal health and physical fitness. These qualities have become of the highest national importance, and their value is being made evident in the eyes of all. The occasion, as individuals and households are discovering, is not one merely for admiring skill and vigour in others, but for the cultivation of these characteristics in ourselves. We believe this lesson to be a useful one for the nation even when considered merely in relation to the public health, and we trust its influence will continue long after the present war has passed into history. Whether the means now in operation are to be continued or not involves political issues which are not for discussion here. But it is reasonable to hope that present experiences will have heightened public appreciation both of the value of bodily fitness and of the duty of the individual to cultivate it, and this alike for his own sake and for the sake also of the community to whom he owes some obligation of service. From the unmeasurable evils of the war there will thus be drawn something of value to the life and vigour and worth of the whole nation.

So far we have spoken of physical fitness, as one of our cares here is the public health. We by no means undervalue from the same point of view the mental and moral forces now at work in the country, though of these this is hardly the place to speak at length. The stimulus of a great purpose, and the courage and resolution to fufil it, the shrinking of petty aims and sordid motives, and the sense of union and co-operation have their play and action even in the physical sphere. They help towards the growth of a robust manhood and a robust nationhood, and though the occasion which brings them into operation is an unhappy one, they themselves are wholly beneficial and helpful.

Another outcome of the war which we may welcome is an increased appreciation of the national importance of doctors and nurses and hospitals. The relation of these to an army in the field is being displayed in a manner both forcible and pathetic. Amid the stress of our anxiety it is much to know that our husbands and brothers and sons have the certainty of efficient medical care and nursing should they be called on to suffer in defence of their country. Even at the sacrifice of their own lives our Army doctors have in not a few instances shown that those who fall on the field of battle shall not be left without succour; and there is the most direct testimony that neither skill nor kindliness is wanting in the treatment and nursing of the wounded. It is impossible that all this can be of no effect. Indeed, the widespread desire to be associated with such efforts, whether by gifts of money or by personal service, is proof that the country generally has been roused to a sense of the large part that medical and nursing efficiency play in the success of the struggle in which the nation is engaged.

Lastly, we wish to notice the conspicuous position in which the war has placed our voluntary hospitals. Without these there would indeed have been an enormous gap in the arrangements necessary for the adequate care of the wounded soldiers sent home from the Front. In all parts of the country wards and beds have been placed freely at the disposal of the authorities, and it is perhaps to be feared that this has sometimes meant some prejudice to the needs of the civil population. There has, however, been universal good will, and everyone concerned has been ready to make sacrifices and to suffer inconveniences so that the sick and wounded soldiers may have what they so well deserve. The public, we are sure, will not fail to remember that in a time of great national emergency our hospital governors and hospital staffs freely placed their great resources at the disposal of the country. The attention thus attracted to these institutions will, we hope, lead to a realisation of the truth that the ordinary work of the hospitals, equally with the special duties of to-day, is service rendered to the nation, and thus once again the evils of war may be associated with an education which is wholly beneficial and helpful to the community.

